# Proteomic Profiles of Mesenchymal Stem Cells Induced by a Liver Differentiation Protocol

**DOI:** 10.3390/ijms11124905

**Published:** 2010-11-30

**Authors:** Kawin Leelawat, Siriluck Narong, Suthidarak Chaijan, Khanit Sa-ngiamsuntorn, Sinee Disthabanchong, Adisak Wongkajornsilp, Suradej Hongeng

**Affiliations:** 1 Department of Surgery, Rajavithi Hospital, Rajathevi, Bangkok, 10400, Thailand; E-Mails: kawin.leelawat@gmail.com (K.L.); sirilucknarong@hotmail.com (S.N.); bubu_b600@hotmail.com (S.C.); 2 Department of Surgery, Faculty of Medicine, Rangsit University, Bangkok, 10400, Thailand; 3 Department of Pharmacology, Faculty of Medicine, Siriraj Hospital, Mahidol University, Bangkok, 10400, Thailand; E-Mail: khanits@hotmail.com (K.S.); siawj@mahidol.ac.th (A.W.); 4 Department of Internal Medicine, Faculty of Medicine, Ramathibodi Hospital, Mahidol University, Bangkok, 10400, Thailand; E-Mail: sineemd@hotmail.com; 5 Department of Pediatrics, Faculty of Medicine, Ramathibodi Hospital, Mahidol University, Bangkok, 10400, Thailand

**Keywords:** human mesenchymal stem cells, liver differentiation, proteomic study

## Abstract

The replacement of disease hepatocytes and the stimulation of endogenous or exogenous regeneration by human mesenchymal stem cells (MSCs) are promising candidates for liver-directed cell therapy. In this study, we isolated MSCs from adult bone marrow by plastic adhesion and induced differentiation with a liver differentiation protocol. Western blot analyses were used to assess the expression of liver-specific markers. Next, MSC-specific proteins were analyzed with two-dimensional (2D) gel electrophoresis and peptide mass fingerprinting matrix-assisted laser desorption/ionization (MALDI)-time of flight (TOF)-mass spectrometry (MS). To confirm the results from the proteomic study, semi-quantitative reverse transcription-polymerase chain reaction (RT-PCR) analyses were performed. We demonstrated that MSCs treated with the liver differentiation protocol expressed significantly more albumin, CK19 and CK20, than did undifferentiated cells. In addition the results of proteomic study demonstrated increases expression of FEM1B, PSMC2 and disulfide-isomerase A3 in MSCs treated with the liver differentiation protocol. These results from proteomic profiling will not only provide insight into the global responses of MSCs to hepatocyte differentiation, but will also lead to in-depth studies on the mechanisms of proteomic changes in MSCs.

## 1. Introduction

Most liver diseases lead to hepatocyte dysfunction, with the possibility of eventual organ failure. Previous studies demonstrated the plasticity of MSCs to differentiate into other cells of the mesodermal lineage, including pancreatic cells and hepatocyte-like cells *in vitro* [1–3]. Therefore, the replacement of disease hepatocytes and the stimulation of endogenous or exogenous regeneration by human mesenchymal stem cells (MSCs) are promising candidates for liver-directed cell therapy. Previous studies have demonstrated that MSCs possess an extensive potential to differentiate into hepatocyte-like cells by using cytokines and growth factors that have a potent effect on hepatic growth and differentiation *in vitro* [4]. However, the effect of this protocol on MSCs has not been investigated comprehensively, and should be addressed before clinical use.

With conventional molecular biological assays, the identification of protein expression can be performed on a limited number of proteins. Proteomics offer a systematic study of the quantitative and qualitative mapping of the entire proteome [5]. In this study, we induced MSCs using a liver differentiation protocol and two-dimensional (2D) electrophoresis to separate the proteins by isoelectric focusing (IEF) and SDS-PAGE. Proteins separated by 2D electrophoresis were then digested and analyzed by mass spectrometry (MS). We demonstrated that increased expression of the mesenchymal marker vimentin and FEM1B, PSMC2 and disulfide-isomerase A3 were found in MSCs treated with the liver differentiation protocol.

## 2. Results

### 2.1. Induction of MSCs Using the Liver Differentiation Protocol

**Figure 1.**
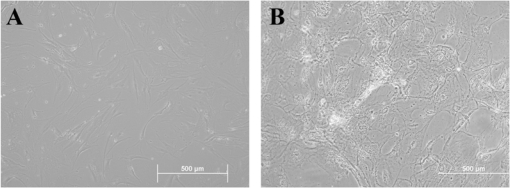
The MSCs cells culture under a phase contrast microscope at 20 × magnification: (**A**) Control; (**B**) cells were treated with liver differentiation protocol for 28 days.

**Figure 2.**
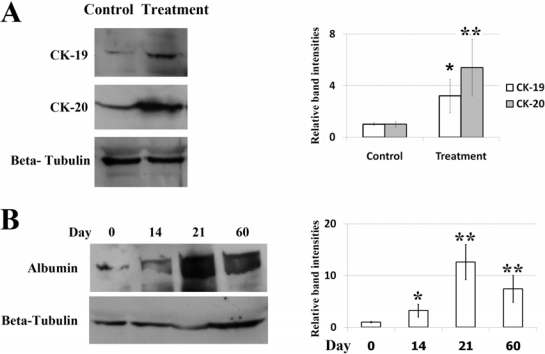
(**A**) The expression of CK-19 and CK-20 in MSCs cells was determined by western blotting. Cells were treated with liver differentiation protocol or control vehicle (DMSO) for 21 days. *β* tubulin was used as a loading control. The relative levels of CK-19 and CK-20 protein were assessed by scanning densitometry of western blots. Data represent the mean ± SD of three independent experiments (significant as compared with control, * *p* = 0.04, ** *p* < 0.001); (**B**) The expression of albumin in MSCs cells treated with liver differentiation protocol for 0–60 days was determined by western blotting. *β* tubulin was used as a loading control. The relative levels of albumin protein were assessed by scanning densitometry of western blots. Data represent the mean ± SD of three independent experiments (significant as compared with control, * *p* = 0.002, ** *p* < 0.001).

**Figure 3.**
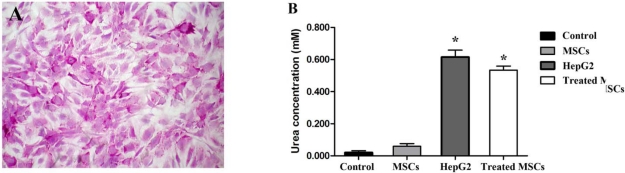
(**A**) Detection of glycogen in the cytoplasm of MSCs treated with liver differentiation protocol was demonstrated by PAS staining. PAS positive substances stain pink in the cytoplasm of the cells; (**B**) The urea production was determined. Cells were treated with liver differentiation protocol or control vehicle (DMSO) for 21 days. Studies were done in triplicate and repeated twice. Data represent the mean ± SD of three independent experiments (significant as compared with control, * *p* < 0.001).

As revealed by morphological studies, MSCs cultured in the liver differentiation media for four weeks adopted polygonal cell morphology. The nucleus and cytoplasm appeared granulated (Figure 1) and were still viable after 60 days. To verify whether these differentiated cells had the characteristic expression of hepatic phenotypic markers, protein from undifferentiated and differentiated cells was extracted. Western blot analyses demonstrated that differentiated MSCs expressed significantly more albumin, CK19 and CK20 than did undifferentiated cells (control) (Figure 2A). In addition, the expression of albumin was also found in differentiated MSCs after 60 days (Figure 2B). The presence of glycogen in the cytoplasm of differentiated MSCs was demonstrated by PAS staining (Figure 3A). Additionally, MSCs themselves did not produce urea. When MSCs were cultured for three weeks in the liver differentiation media, urea was secreted in the supernatant (Figure 3B).

### 2.2. Protein Expression Changed in Differentiated MSCs

**Figure 4.**
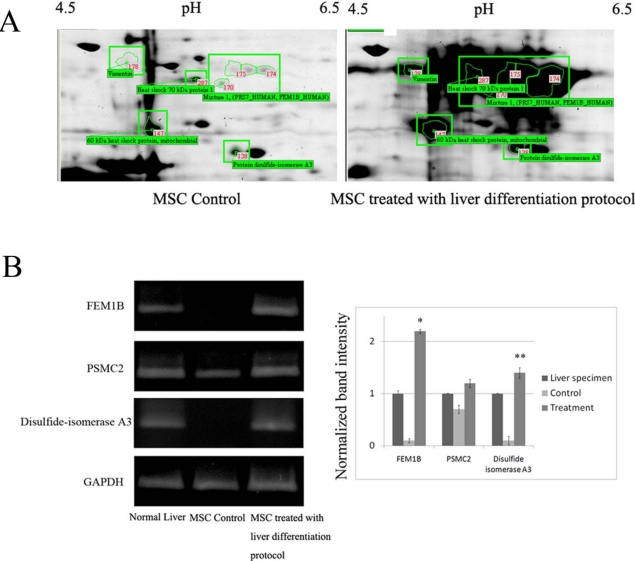
(**A**) Proteome map of protein extraction from MSC control and treatment. Identification of proteins were performed by 2D gel electrophoresis follow by MALDI-TOF-MS. Isoelectric focusing were performed with 50 μg total proteins from MSC using 3–10 pH-strips, 18 cm. SDS-PAGE were performed on 12.5% gels and stained with Coomassie blue G-250; (**B**) Total RNA was isolated from MSCs cells treated with or without liver differentiation protocol and liver specimens. Expression of FEM1B, PSMC2 and disulfide-isomerase A3 mRNA levels was evaluated using RT-PCR. The results, based on the ratio of these mRNA amplification to that of GAPDH, are presented as the fold increase relative to the mRNA levels from liver specimens. The results are expressed as the mean ± SD of three separate experiments (* *p* < 0.001, ** *p* = 0.01 *versus* the control MSC).

We used a proteomic approach to profile the protein expression in MSCs treated with the liver differentiation protocol. The proteins in the cell lysates were separated by two-dimensional electrophoresis followed by Coomassie staining. A representative two-dimensional gel image of protein lysates from MSCs with and without being cultured in the liver differentiation protocol is shown in Figure 4A. These protein spots, encompassing a wide range of molecular weights, pI values, and abundance were resolved and identified with high confidence (95%). We demonstrated that increases in vimentin, FEM1B, PSMC2 and disulfide-isomerase A3 expression were found in MSCs treated with the liver differentiation protocol.

To confirm the results of the proteomic studies, total RNA from undifferentiated and differentiated MSCs cells and normal liver specimens was isolated, and the mRNA levels of the genes of interest were examined by RT-PCR. Expression of FEM1B, PSMC2 and disulfide-isomerase A3, were significantly increased in these differentiated cells when compared to the undifferentiated cells (Figure 4B).

**Figure 5.**
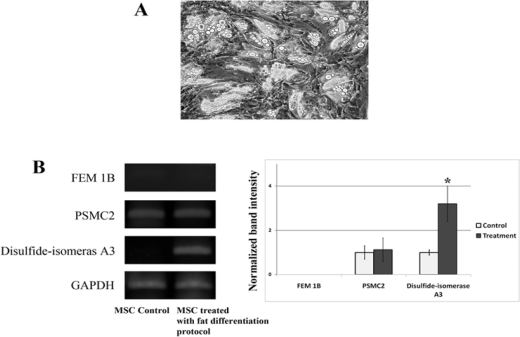
(**A**) The fat globules were demonstrated in the cytoplasm of MSCs cells treated with fat differentiation protocol; (**B**) Total RNA was isolated from MSCs cells treated with or without fat differentiation protocol. Expression of FEM1B, PSMC2 and disulfide-isomerase A3, mRNA levels was evaluated using RT-PCR. The results, based on the ratio of these mRNA amplification to that of GAPDH, are presented as the fold increase relative to the mRNA levels from MSC control. The results are expressed as the mean ± SD of three separate experiments (* *p* < 0.001 *versus* the control MSC).

To study whether or not the expression of FEM1B, PSMC2 and disulfide-isomerase A3 was specific only for MSCs treated with the liver differentiation protocol, we measured the expression of these genes in the MSCs treated with fat differentiation protocol. These MSCs treated with fat differentiation protocol were derived from our previous study [6]. These cells demonstrated many fat globules in their cytoplasm (Figure 5A). The results demonstrated that the expression of disulfide-isomerase A3 was up-regulated in MSCs treated with fat differentiation protocol. However, there is no change in the expression of FEM1B and PSMC2 in MSCs treated with fat differentiation protocol when compared to the undifferentiated cells (Figure 5B).

## 3. Discussion

Several studies have demonstrated that MSCs easily differentiate into mesenchymal tissue lineages, including bone, cartilage, fat, tendon, muscle, and bone marrow. Moreover, they also differentiate into cells of ectodermal origin, such as neurons [7]. Our findings demonstrate that treating MSCs with the liver differentiation protocol induces a profound change in gene and protein expression that resembled cells derived from hepatocytes from the endoderm. These findings were reproducible in cells from six donors of both sexes.

The present study demonstrates that MSCs treated with the liver differentiation protocol expressed proteins that are compatible with liver cells, including albumin, CK19 and CK20. This finding was further supported by the positive results of PAS staining and urea production. Using two-dimensional gel electrophoresis (2-DE) and mass spectrometry (MS), we demonstrated for the first time that PSMC2, FEM1B and disulfide-isomerase A3 were highly expressed in MSCs treated with the liver differentiation protocol. We also found that the expression of PSMC2 and FEM1B was demonstrated in MSCs treated with the liver differentiation protocol but not in MSCs treated with the fat differentiation protocol. PSMC2 is the 26S proteasome. that is composed of two complexes, a 20S core and a 19S regulator. Functionally, the PSMC2 gene has been proposed to participate in protein catabolic processes or proteolysis [8]. Previous studies demonstrated that this PSMC2 gene can be detected at high levels in injured skeletal muscle [9], and liver [8]. This finding suggested that the up-regulation of PSMC2 was associated with an increase of protein catabolic processes in MSCs treated with the liver differentiation protocol.

PDIA3 (protein disulfide isomerase A3) is a member of the endoplasmic reticulum stress signaling pathway, and its expression level increases in response to cellular stress due to its function as a chaperone. It interacts with the lectin chaperones. calreticulin and calnexin. to modulate the folding of newly synthesized glycoproteins [10]. PDIA3 was also up-regulated in MSCs treated with the liver differentiation protocol. Previous studies demonstrated that PDIA3 was strongly expressed in many kinds of cells, including pancreatic, placental, lung and liver cells [11]. Recently published data connected PDIA3 to apoptosis and demonstrated an anti-apoptotic effect of PDIA3 in the melanoma cell line A375 after induction of ER stress [12]. In this study, we also demonstrated that PDIA3 is up-regulated in MSCs treated with fat differentiation protocol. Therefore, we hypothesize that the observed increase of PDIA3 in this study is most likely due to elevated cellular stress.

A previous study has demonstrated that *fem-1* is an ankyrin repeat protein involved in the regulatory pathway controlling cell fate decisions during sex determination in the nematode [13]. The *Fem1* gene family that encodes *fem-1* homologs has been characterized and consists of at least three members in the mouse, designated *Fem1a*, *Fem1b*, and *Fem1c*; these *Fem1* members have highly conserved homologs in humans, designated FEM1A, FEM1B, and FEM1C, respectively [14]. The mammalian *Fem1* genes are expressed in tissues involved in glucose physiology, including pancreatic tissue and skeletal muscle [13]. In this study, we found that human liver tissues and MSCs treated with the liver differentiation protocol expressed FEM1B. We suggested that the up-regulation of FEM1B may be involved in the metabolism of glucose in MSCs treated with the liver differentiation protocol. The exact role of FEM1B in human liver cell function and differentiation should be further- investigated.

## 4. Experimental Section

Dulbecco’s Modified Eagle’s medium (DMEM-low glucose) was purchased from Gibco (Paisley, U.K.). Phosphate-buffered saline (PBS) and dexamethasone were obtained from Merck Pharma (Mollet del Vallés, Spain). Trypsin-EDTA and newborn calf serum were obtained from Biochrom AG (Berlin, Germany). ITS and premix were purchased from BD Biosciences (Madrid, Spain). Basic fibroblast growth factor (bFGF) was purchased from Invitrogen (Barcelona, Spain). Hepatocyte growth factor (HGF), epidermal growth factor (EGF) and nicotinamide were from Sigma-Aldrich (Madrid, Spain). Oncostatin M (OMS) was purchased from PeproTech EC (London, U.K.). Polyclonal rabbit anti-human albumin, alphafetoprotein, polyclonal goat anti-mouse HRP and polyclonal goat anti-rabbit HRP were purchased from DakoCytomation (Barcelona, Spain).

### 4.1. Isolation and Culture of MSCs

MSCs were selected from 10 mL aspirates from the iliac crest of normal adult donors after informed consent was given. Cells were plated at a density of 1,000,000 cells per 25 cm^2^ flask in 5 mL of DMEM-low glucose supplemented with 100 μg/mL penicillin, 100 μg/mL streptomycin (Invitrogen), 2 mmol/L l-glutamine (Invitrogen), and 10% fetal calf serum (Invitrogen), incubated at 37 °C in a humidified atmosphere containing 5% CO_2_. After 72 hours, the nonadherent cells were discarded, and adherent cells were washed gently with medium and cultured for approximately 21 days. Fresh complete medium was replaced twice a week.

### 4.2. Liver Differentiation Protocol

MSCs cultured at 85% confluency were used for the differentiation assays. The cells were cultured in DMEM supplemented with 20 ng/mL EGF and 10 ng/mL bFGF. Then a 2 step differentiation protocol was performed. First, the cells were cultured in step-1 differentiation medium consisting of DMEM supplemented with 20 ng/mL HGF, 10 ng/mL bFGF and 4.9 mmol/L nicotinamide, for 7 d, followed by culture in step 2 differentiation medium that consisted of DMEM supplemented with 20 ng/mL OMS, 1 mmol/L dexamethasone, and 10 mL/mL ITS + premix (final concentration: 100 mmol/L insulin, 6.25 mg/mL transferrin, 3.6 mmol/L selenious acid, 1.25 mg/mL BSA and 190 mmol/L linoleic acid) to achieve cell maturation (up to Day 21).

### 4.3. Western Blot Analyses

Control MSCs and MSCs treated with the liver differentiation protocol were collected, washed with PBS and lysed in lysis buffer. Western blot analyses were performed as previously described [16]. The blots were first probed with antibodies against albumin, cytokeratin 19 and cytokeratin 20 and then reprobed with antibodies against tubulin. Bound antibodies were detected using chemiluminescence.

### 4.4. Periodic Acid-Schiff

Periodic acid-Schiff (PAS) staining was performed to detect glycogen granules in the hepatocytic cytoplasm, according to the manufacturer’s instructions (Sigma). The cells were fixed in formalin-ethanol fixative solution for 1 minute at room temperature. Then the slides were immersed in periodic acid solution for 5 minutes and Schiff’s reagent for 15 minutes. Counterstain slides in Hematoxylin solution and examined under the light microscope.

### 4.5. Urea Production Assay

Urea concentrations were determined by colorimetric assay (QuantiChromTM Urea Assay Kit) per manufacturer’s instructions. Briefly, 20 μL of standard, culture supernatant or media was added to a cuvette; then 1 mL of working reagent was added and incubated for 20 minutes before the absorbance reading at the optical density at 520 nm was examined. HepG2 (human hepatocellular carcinoma cell line) grown in monolayer were used as a positive controls and fresh culture medium used as negative control.

### 4.6. Two-Dimensional Gel Electrophoresis (2D-GE), Gel Scanning and Image Analysis

For the first dimension, the protein samples were combined with the rehydration buffer (7 M urea, 2 M thiourea, 4% CHAPS, 60 mM DTT, 1% Pharmalyte or IPG Buffer pH 3–10) to a total volume of 340 mL. An isoelectric focusing gel was performed using an 18 cm Immobilime DryStrip gel with a linear pH 3–10 and an Ettan IPGphor 3 Manifolds system (GE Healthcare, U.S.) for a total of 32 kVh at 20 °C. Following IEF, each strip gel was equilibrated with equilibration buffer. The strip gels were loaded and run in 12.5% acrylamide gels using the Ettan DALT*six* Electrophoresis System. The run was stopped once the bromophenol blue dye front had run off the bottom of the gels. The gels were stained with Colloidal Coomassie staining, and the proteins were visualized using ImageScanner. The gel images were analyzed using a differential protein expression profile by ImageMaster 2D Platinum software (GE Healthcare).

### 4.7. Tryptic in-Gel Digestion of 2-DE Spots and MALDI-TOF MS

Protein spots were picked and transferred into a 96-well microplate and subjected to in-gel digestion, which was performed using an Ettan Spot Handling workstation (GE Healthcare). Peptide mass fingerprinting (PMF) was performed using an Autoflex MALDI-TOF mass spectrometer (Bruker Daltonics, Germany). The matrix solution, a-cyano-4-hydroxycinnamic acid (Bruker), was prepared by dissolving one part acetonitrile with two parts 0.1% trifluoroacetic acid in water. The digested peptide was then mixed with matrix solution, and the mixture was spotted onto a stainless-steel target and left to dry at room temperature. The PMFs were recorded by reflectron mode. The resulted PMFs were visualized by flexAnalysis software version 2.0 (Bruker Daltonics).

### 4.8. Database Search for Protein Identification

PMFs were used for protein identification from tryptic fragment sizes using the MASCOT search engine (www.matrixscience.com) based on the entire NCBInr protein database (Homo sapiens) using peptide monoisotopic peaks.

### 4.9. RNA Extraction and RT-PCR

Total RNA was extracted from undifferentiated (used as control) and differentiated cells using Trizol reagent. Detection of mRNA transcription of FEM1B, PSMC2, disulfide-isomerase A3 and GAPDH were performed by RT-PCR. The sequences of both the forward and reverse primers and the expected sizes of the PCR-amplified DNA are listed in Table 1. Amplification and detection was performed in a BIO-RAD iCycler iQ system (Bio-Rad, Hercules, CA). The reaction conditions were reverse transcription at 60 °C for 10 min, followed by the amplification by initial denaturation at 95 °C for 30 s and 40 cycles of denaturation at 95 °C for 30 s, annealing at 60 °C for 30 s, and extension at 72 °C for 60 s, respectively. The PCR products were detected with 1.5% agarose gel electrophoresis.

### 4.10. Statistical Analysis

The experiments were all performed in triplicate, and each result is reported as the mean plus or minus the SD. The data between the two groups were compared using the Student’s *t*-test. A *p*-value of less than 0.05 was considered as statistically significant.

## 5. Conclusions

In summary, this study identified a set of proteins that were up-regulated in MSCs treated with a liver differentiation protocol. These proteins were involved in important functional processes important for such cellular functions as apoptosis, protein folding, and metabolism. We believe that these observations shed new light on the processes of liver differentiation from MSCs.

## Figures and Tables

**Table 1.** Primer Sequences for RT-PCR.

**Primer**	**Forward**	**Reverse**	**Length**
FEM1B	ACATTACCGGGTGCAGACTC	TTGTTGGCAATGCTGATGTT	255 bp
PSMC2	GGCAGATCAAGCAAGTTGAAG	TATTTTGGGTCCTCCGAATC	205 bp
PDIA3	CGAGCGCAAGCAGCGGGTTA	TGTCCACACCAGGGGGCGAA	271 bp
GAPDH	GAAGGTGAAGGTCGGAG	GAAGATGGTGATGGGATTTC	226 bp
